# Core policies disparity response to COVID-19 among BRICS countries

**DOI:** 10.1186/s12939-021-01614-z

**Published:** 2022-01-20

**Authors:** Jun Jiao, Leiyu Shi, Yuyao Zhang, Haiqian Chen, Xiaohan Wang, Manfei Yang, Junyan Yang, Meiheng Liu, Gang Sun

**Affiliations:** 1 Department of Health Management, School of Health Management, Southern Medical University, Guangzhou, Guangdong 510515 P.R. China; 2 Department of Health Policy and Management, Bloomberg School of Public Health, Johns Hopkins University, Baltimore, MD 21205 USA

**Keywords:** COVID-19, BRICS health equity, National response, Core strategy comparison

## Abstract

**Objective:**

To provide experience for formulating prevention and control policies, this study analyzed the effectiveness of the Coronavirus disease 2019(COVID-19) prevention and control policies, and evaluated health equity and epidemic cooperation among BRICS countries.

**Methods:**

This study summarized the pandemic prevention and control policies in BRICS countries and evaluated the effectiveness of those policies by extracting COVID-19 related data from official websites.

**Result:**

As of May 4, 2021, responding to COVID-19. China adopted containment strategies. China’s total confirmed cases (102,560) were stable, without a second pandemic peak, and the total deaths per million (3.37) were much lower than others. India and South Africa who adopted intermediate strategies have similar pandemic curves, total confirmed cases in India (20,664,979) surpassed South Africa (1,586,148) as the highest in five countries, but total deaths per million (163.90) lower than South Africa (919.11). Brazil and Russia adopted mitigation strategies. Total confirmed cases in Brazil (14,856,888) and Russia (4,784,497) continued to increase, and Brazil’s total deaths per million (1,936.34) is higher than Russia (751.50) and other countries.

**Conclusion:**

This study shows BRICS countries implemented different epidemic interventions. Containment strategy is more effective than intermediate strategy and mitigation strategy in limiting the spread of COVID-19. Especially when a strict containment strategy is implemented in an early stage, but premature relaxation of restrictions may lead to rebounding. It is a good choice to combat COVID-19 by improving the inclusiveness of intervention policies, deepening BRICS epidemic cooperation, and increasing health equities.

## Introduction

In Dec 2019, the first Viral Pneumonia of Unknown Cause was reported in Wuhan, Hubei province, China [1]. The pandemic rapidly spread worldwide, on Jan 30, 2020, the World Health Organization (WHO) declared the Coronavirus disease 2019(COVID-19) outbreak a public health emergency of international concern [2]. Based on national health care system, population structure, and epidemic situations, countries have taken different interventions, including restrictive measures, border prevention and control measures, health protection measures, and key population control measures. Countries are also trying to achieve herd immunity through vaccines. USA, Russia and China successfully developed vaccines and carried out mass vaccination. However, due to the limitations of research, immunization duration, and other factors, it needs a long time to achieve herd immunity [3]. Furthermore, there were 3,815 coronavirus genotypes [4], and coronavirus variants are still increasing [5]. In this case, social intervention policies remain necessary to combat COVID-19.

With the deepening globalization, on Mar 11, 2020, WHO Director-General, Dr. Tedros declared COVID-19 was a global pandemic [6], which needs the world to work together. However, vaccine nationalism, racial discrimination, health resources differences, inadequate primary health care system, and barriers of health products trade hampered health equity and COVID-19 improvement. In this study, we choose BRICS countries as our study subjects, which are made up of five major emerging countries with global influence [7], including Brazil, Russia, India, China, and South Africa. Their whole population is more than 3 billion. Their policies and effectiveness have influenced world COVID-19 pandemic. Responding to COVID-19, developed countries failed to play a leading role, nor can lead the global economic recovery [8]. Since established, BRICS countries have been carrying out public health cooperation and making efforts for health equity.

Containment and mitigation strategies, as guiding strategies for different phases of influenza pandemics, were first proposed in the WHO Global Influenza Preparedness Plan in 2005 [9]. Containment strategy aims to prevent spread of infection in defined areas [10], which prefers social intervention measures to rapidly block spread of epidemics in an early stage [11]. In fact, main points of containment strategy were reflected in SASR 2004 prevention and control measures, and were more fully implemented in H1NI 2009 prevention and control measures. Some scholars suggest that measures based on isolation are the fundamental anti-pandemic measures [12]. However, some scholars believe that it is difficult to implement, especially at the epidemic peak to track cases [13]. In COVID-19, scholars generally believe that containment strategy can help control spread and reduce mortality [14], and key measures including lockdown, isolation and tracing of close contacts play an important role [15–17]. However there are different opinions about stages and scope of containment strategy, Hyunjin Son and Silvia Caristia respectively believe that containment strategy is more suitable in limit scopes and early pandemic stages [18, 19]. Mitigation strategy is recommended for actions in phase 5 and 6 of pandemic, essentially reducing the pandemic impact [10]. Intermediate strategy came into being in the discussion of implementation stage of containment and mitigation strategy. Proponents of an intermediate strategy believe that containment and mitigation strategy should be taken at different epidemic stages. However, they have different opinions on the timing of changing the response strategy. Factors including clinical and epidemiological characteristics of a new virus and the impact of control measures early a pandemic should be analyzed in real time [20–22]. Mathias Peirlinck proposed to shift to mitigation strategy induced by behavior changes when asymptomatic carriers become at high risk [23]. Mitigation strategy supporters based on conditions that it is almost impossible to fully manage infection source because of incubation period and the asymptomatic. In that way, epidemic can’t be blocked. It should be allowed to spread in a manageable way until forms an immune barrier. Krishna Regmi supported mitigation strategy might be the way forward and more acceptable [24]. Benjamin J Cowling believed social distancing has a less disruptive social and economic impact than complete lockdown, and also can meaningfully control COVID-19 with a wider range of application [25].

With COVID-19 spread, there were differences in response policies and effectiveness among BRICS countries. We studied COVID-19 epidemic prevention and control policies among BRICS countries, including China adhered to containment strategy, which focusing on the whole transmission process. India and South Africa adopted intermediate strategies that took different strategies at different epidemic stages. Brazil and Russia took mild mitigation strategies to ease pressure on health care systems. To provide experience for formulating prevention and control policies, improving health equity among BRICS countries and carrying out international epidemic cooperation, this study analyzed the effectiveness of COVID-19 prevention and control policies and evaluated health equity and epidemic cooperation among BRICS countries.

## Methods

COVID-19 data of this study (total confirmed cases, daily new cases, total deaths) were extracted from official websites, which included COVID-19 daily updates from the National Health Commission, PRC, Johns Hopkins University & Medicine Coronavirus Resource Center and, WHO. COVID-19 pandemic prevention and control policies of BRICS countries were collected from BRICS government web pages, WHO website, and the University of Washington COVID-19 policy information website. Then chronological sorted to form COVID-19 pandemic data chart and time axis of national responses.

## Result

### National response to the COVID-19 pandemic

#### Containment strategy of China

In Dec 2019, China reported COVID-19. After COVID-19 began to spread, China took measures, including lockdown Wuhan city, closing the passage, and asking citizens to quarantine at home on Jan 23, 2020. Public health measures were launched nationwide to manage “four categories of people (confirmed cases, suspected cases, febrile patients who might be carriers, and close contacts)”, follow the “four early” principle, and block transmission route. To guarantee treatment and reduce deaths, government finance covered all costs of COVID-19 treatment. Furthermore, government coordinated national manpower, medical equipment, and materials to support Hubei Province, quickly built 16 mobile cabin hospitals, and organized the province-city medical pairing support relationship. On May 8, 2020, China’s epidemic work moved from emergency status to normalization. Prevention and control policy shifted to import cases prevention, sporadic cases containment, epidemic normalization prevention, and promotion of scientific and technological breakthroughs in vaccines and drugs. Table 1 summarizes the main containment measures in China.

**Table 1:** The three-stage major measures taken for COVID-19 in China

Stage	Measures
Stage 1: Initial stage of COVID-19	1 Classification of infectious diseases: the COVID-19 included in category B infectious diseases, and adopted prevention and control measures for Category A infectious diseases. 2 Established COVID-19 Command System: (1) The National Health Commission made arrangements to send expert teams to Wuhan to conduct on-site investigations. (2) The central government set up a leading group on the disease response.
Stage 2: spreading stage of COVID-19	1 Lockdown Wuhan city: (1) Activated public health emergency II level responses of Hubei province. (2) On Jan 23, 2020: temporarily closed Wuhan outbound routes of airports and railway stations at 10 a.m.; Suspended city's passenger transport operations; People were told to cannot leave Wuhan and home quarantine; Lockdown Wuhan city lasted 76 days. 2 Community and social control measures: (1) All provinces activated Level 1 public health emergency response. (2) Adopt measures to put “four categories of people” under classified management and conducted mass screenings to search for them; All those in need are tested, isolated, hospitalized, or treated was implemented. (3) Strictly observed the principle of early detection, reporting, quarantine, and treatment. (4) Extended the Chinese New Year holiday of 2020 and postponed the opening of schools. (5) Required residents to implement home quarantine, 14-day isolation after trans-regional travel, and protective measures including wearing masks, maintaining social distance and reducing gatherings. 3 Medical treatment policy: (1) Medical expenses incurred by COVID-19 patients will be covered by basic medical insurance, critical illness insurance and medical assistance, and the individual portion will be subsidized by the government. Medical insurance has also reduced the cost of treatment for suspected patients. (2) Established a mechanism to organize pairing assistance from other provinces to Hubei’s cities for treatment; Nationwide resources were mobilized to assist Hubei province; Rapidly constructed 16 mobile cabin hospitals by mobilizing nationwide medical equipment. (3) Produced and released Diagnosis and Treatment Protocol for Novel Coronavirus Pneumonia, which adding methods of traditional Chinese medicine.
Stage 3: normalize prevention and control stage	1 Guard against imported cases: (1) Tighten up border management, implemented "the first point of entry management" and nucleic acid testing of inbound personnel. (2) Implemented the declaration of health information for inbound personnel, and suspended the entry of foreigners holding Chinese visas and residence permits. 2 Prevent a rebound in indigenous cases: (1) Adopted precise and differentiated epidemic control strategies: Low-risk regions should focus on imported cases and restore production and life at an appropriate time; Medium-risk regions should prevent both imported cases and indigenous spread; High-risk regions should strictly commit epidemic prevention and control. (2) Using health color code as identification and the evidence of daily life and access to public places. (3) For sporadic cases: Strengthen screening of close contacts; Trace source of confirmed cases; Strengthen early-warning research and risk grading; Strengthen quarantine control of key personnel. 3 Expedited vaccines and medicines research and application: (1) COVID-19 therapeutic drugs were included in new scope of medical insurance list. (2) In Dec, launched a key population vaccination campaign.

#### Intermediate strategy of India and South Africa

On Jan 30, 2020, India reports the first confirmed case. Before that, India implemented immigration control measures to block import cases, and restricted medical supplies and medicines export to ensure domestic needs. From Mar 25 to June 30, 2020, India entered a five-phase “lockdown” with regional management. Then, India began to stage unseal, enterprises gradually reopened. On Sep 1, 2020, India became the world’s fastest country of daily new cases, then some cities restarted public health measures. In Feb 2021, India relaxed social control, held traditional cultural festivals, and almost completely abandoned social distance policies.

South Africa is far away from the Eurasian continent. Until Mar 5, 2020, South Africa reported the first case imported from Italy. Then government began to take measures, such as closing borders and ports, strengthening epidemic surveillance. Midnight on Mar 26, 2020, South Africa began a 21-day lockdown as the strictest Level 5. On April 23, 2020, government gradually reduced blockade levels, relaxed community control measures, implemented “risk adjustment strategy”, and carried out an economic policy of resuming work and production. When COVID-19 epidemic rebounded with a second outbreak, border control, social prevention and control measures such as curfews and masks were reintroduced, but the country’s block level was not changed. In contrast to other countries’ eagerness to vaccine, South Africa moved slowly, opened vaccination registry on April 16, 2021. Table 2 summarizes the main epidemic control measures in India and South Africa that combine containment and mitigation policies.

**Table 2:** The four-stage major measures taken for COVID-19 in India and South Africa

Country	India	South Africa
Stage		
Stage 1: Prevention and control of import cases	1 Immigration Control Measures: (1) On Jan 25, 2020, issued a travel warning that avoiding unessential travel to China. (2) On Feb 2, stopped the electronic visa service for Chinese; On 13 Mar, all travel visas for foreigners became temporarily invalid except special visa categories. (3) Closed the border and banned international flights. (4) Passengers from severely affected countries would be under quarantine for 14 days. 2 Guaranteeing epidemic prevention materials: (1) Since Jan 31, the export of personal protective equipment including masks and protective clothing was banned during the epidemic. (2) Banned the export of 26 active pharmaceutical ingredients (APIs) and their medicines.	1 Border control measures: (1) Closed country borders; Cross-provincial travel is prohibited. (2) Closed 35 land ports and 2 waterways; Strengthened airport monitoring measures. (3) Suspended visa issuance to visitors from high-risk countries such as China and Italy and foreigners who have visited these countries in the past 20 days. (4) Installed temperature monitoring equipment at all ports; designated 13 public hospitals across the country to treat COVID-19 patients and provided free treatment. 2 On Mar 15, 2020, South Africa entered into a state of national disaster and started a crisis management mechanism.
Stage 2: spreading stage of COVID-19	1 Implement a five-stage blockade: Lockdown-1 imposed a nationwide strict lockdown and isolation for 21 days; closed all shops, factories, and so on; Suspended all unessential activities. Lockdown-2 divided hotpots and non-hotpots region, which relaxed restrictions, allowed some necessary activities, and gradually resumed work and production. Lockdown-3 divided three zones: red zones were under strict control, orange zones implemented strict residence rules and green zones allowed more free travel. Lockdown-4 imposed curfews and banned going outside except for necessary needs. Lockdown-5 shortened curfews and continued lockdown-4 policy is strictly policed areas, while non-strictly policed areas allowed free movement within the areas, and opened commercial centers. 2 Community prevention and control measures: (1) On Mar 22, imposed a curfew calling on all Indians to stay indoors between 7 and 21. (2) Several states, including New Delhi and Mumbai, made it mandatory to wear masks or be arrested and jailed for at least six months. (3) The government advised Work From Home Policy and pay wages as usual. 3 Guaranteed isolation and treatment sites, and strengthened testing: (1) Retrofitted about 20,000 train carriages for COVID-19 patients’ isolation; Set up nearly 60,000 isolation beds. (2) On Jun 27, built a square cabin hospital in the Bangalore International Exhibition Centre and closed on Sep 4. (3) Enhanced nucleic acid testing in New Delhi.	1 Strictly enforce the blockade order: (1) At midnight on Mar 26, South Africa began a 21-day “lockdown”, and the lockdown level was set as Level 5, the strictest, closed all government offices, businesses, restaurants, and shops, except for public services such as hospitals and pharmacies. (2) Banned all social activities; only allowed people to go out to buy food, medicine, and take medical treatment. (3) Banned sales of alcohol and tobacco; Imposed daily curfew. 2 Implement single room policy: 5,571 isolation sites were set up to provide temporary shelters for the homeless and free self-isolation places for those who cannot self-isolate in their homes. 3 Fighting corruption in COVID-19: The President set up a special commission of inquiry to investigate corruption in the procurement of supplies for the COVID-19 epidemic. 4 Enhance virus detection capability: (1) Public laboratories were working with private laboratories to test for coronavirus. (2) Promoted community detection: Organized medical workers and mobile surveillance vehicles into communities to conduct coronavirus test census, and collected samples of suspected patients. (3) Established community contact tracing teams to track and monitor confirmed patients and close contacts.
Stage 3: Restarting economic and social activities	1 Removing restrictions in stages: In Jun, Unlock-1 opened places of worship, hotels, restaurants, shopping centers all across India and changed curfew time from 21 to 5, while freed cross-border traffic between states. In Jul, Unlock-2 reduced daily curfew by one hour reopened some routes and later resumed some international air travel. In Aug, Unlock-3 opened more activities outside the quarantine zone, while lifting the personal curfew. In Sep, to restore economy Unlock-4 allowed a limited opening of some public facilities, such as subways, shopping malls, and entertainment venues. In Oct, cinema, theatre, and so on were reopened, except the controlled areas, but the number of customers was limited to 50 percent of the authorized capacity; Schools, parks and other places were opened to the public. 2 Economic stimulus policies: (1) Introduced the slogan “India self-made” and launched an economic stimulus package about 20 trillion rupees. (2) Three rounds of economic stimulus: The first round of 20.97 lakh crore, focusing on basic livelihood assistance; The second round focused on stimulating consumption; The third round focused on supporting industries hardest hit by the epidemic, such as tourism and catering.	1 Lower blockade level: (1) From Apr 23, gradually relaxed the level of lockdown order and allowed enterprises to resume work. 2 Relaxed community prevention and control measures: (1) Gradually opened Borders, resumed international travel, relaxed restrictions on public outings and social activities, and allowed most businesses to operate. (2) Relaxed restrictions on the sale of alcohol and tobacco; shortened curfews. 2 Launched economic support policies to restore the economy and stabilize employment: (1) Building a new economy: mobilized public and private resources and undertook massive infrastructure development and maintenance to drive economic recovery. (2) Launched a 500 billion Rand economic support and social relief program. 3 Adjusted the priorities of government work: implement "risk adjustment strategy", enhanced dynamic early warning and epidemic containment measures; Went deep into communities to ensure basic living conditions of residents; Provided support to affected businesses and workers.
Stage 4: COVID-19 second outbreak	1 Public health measures: (1) Mumbai decreed that if there are five or more confirmed cases in a residential building, the entire building and residents will be sealed off. (2) Mass celebrations were banned during the traditional holiday of Holi. (3) New Delhi imposed a daily curfew from 22 to 5. During the curfew, only allowed shops providing essential services and vehicles used for emergency matters. 2 Vaccination measures: (1) On Jan 3, 2021, the emergency use of vaccines was officially approved; Launched vaccination campaign, giving priority to nurses, doctors and other frontline workers. (2) On Mar 25, delayed in Mar and Apr delivery of COVAX, and restricted vaccine exports.	1 Resumed strict community prevention and control measures: (1) Closed borders with neighboring countries such as Zimbabwe, Lesotho and Mozambique. (2) Extended curfews; Asked People to wear masks outside or risk prosecution; Postponed the start of school. (3) Banned on the sale of all alcoholic beverages. 2 Vaccination measures: (1) Implemented a phased vaccine promotion policy: the first phase target medical workers; The second phase is for workers in basic jobs to keep society running, people over 60, with basic medical conditions or in nursing homes; The remaining adults are the target for Phase 3. (2) In the second phase of vaccination, nationwide the number of vaccination sites increased from 53 to 2,000 by collaborating with private medical institutions.

#### Mitigation strategy of Brazil and Russia

In the early stage, Brazilian people and government did not pay attention and only regarded COVID-19 as ordinary flu. In Jan 2020, after receiving reports of COVID-19 suspected cases, Brazil initiated a public health emergency and joint multi-sectoral epidemic prevention team. In the face of the federal government’s lag action, Brazilian states gradually issued epidemic decrees. Many states restricted the flow of people by lockdown city, quarantine measures, and keeping social distance. In June 2020, as many places started economic recovery plans and relaxed quarantine policies, the epidemic worsened in some cities after “being unsealed with epidemic”. Some regions have upgraded prevention and control measures to slow down the COVID-19 outbreak, and vaccination starting on Jan 28, 2021.

On Jan 31, 2020, Russia firstly found two confirmed cases, and government immediately took a series of measures to severely seal border and restrict flow between Chinese and Russian. However, due to Russia’s weak control of immigrants from Europe and North America, and a policy gap, many people from affected areas flowed into Russia. In May 2020, clustering infections were reported in medical institutions and military. In that case, Russian government still implemented a national economic recovery plan. On May 11, 2020, the Russian President announced the end of paid holidays and entered a new stage of fighting COVID-19. Later, prevention and control restrictions were gradually lifted. When the second epidemic began in Oct 2020, states have restarted prevention and control measures, such as restricting gathering, wearing masks, reopening health centers that had been closed in July 2020. Table 3 summarizes the main mitigation measures in Brazil and Russia.

**Table 3:** The four-stage major measures taken for COVID-19 in Brazil and Russia

Country	Brazil	Russia
Stage		
Stage 1: Prevention and control of import cases	1 Public health emergency: (1) On Jan 22, 2020, initiated a public health emergency and joint multi-sectoral epidemic prevention team against COVID-19. (2) On Jan 28, the first suspected case was reported and the health risk level was raised to level 2 (imminent danger) and raised to Level 3 (national public health emergency) in advance. (3) An emergency quarantine law was produced and implemented, and 34 Brazilian nationals were withdrawn from Wuhan and quarantined for 18 days. 2 Early warning: (1) Published a COVID-19 epidemic prevention manual and guidelines. (2) The Brazilian ministry of health and media released real-time information on the epidemic.	1 Established COVID-19 Command System: On Jan 27, 2020, the government meeting on the prevention and control of the spread of the epidemic was held, and an epidemic prevention headquarters was set up. 2 Border prevention and control measures: (1) After the outbreak of the epidemic in China, on Jan 31, Russia closed its far eastern border with China, suspended visa services for Chinese citizens and temporarily banned Chinese citizens from entering the country for private purposes. (2) After the outbreak of the epidemic in Europe, on Mar 18, Russia banned foreign citizens entering the country, stopped issuing tourist visas and required entry quarantine for 14 days. (3) On Mar 27, Russia suspended all international flights.
Stage 2: spreading stage of COVID-19	1 National and local social prevention and control measures: (1) The federal government issued a decree authorizing states to create their quarantine measures. (2) National measures: closed elementary and middle schools; Encouraged people to wear masks of any type and make by themselves. Brazil's health minister ordered a population-wide home quarantine, but the president only supported home isolation for the elderly to protect employment. (3) Local measures: closed schools in cities such as Sao Paulo, Rio and Brasilia; Indefinitely canceled major events; Required to wear masks or be fined; Implemented comprehensive ground lockdown city measures. 2 Border prevention and control measures: on Mar 30, Brazil completely closed its borders. 3 State of public disaster: (1) From Mar 20 to Dec 3, Brazil entered into a state of public disaster. (2) A package of emergency measures: launched R $147.3 billion to deal with the impact of COVID-19 outbreak on economy, and R $4.5 billion to fight COVID-19 directly. 4 Construction of square cabin hospitals: built and operated the Anhembi Square and the Convention Center Square cabin hospital, with a total capacity of 2,000 patients.	1 Set up an epidemic prevention command operation agency and an inter-agency coordination agency. 2 Strengthened surveillance and vaccines: (1) Organized the troop and scientists to strengthen the research on virus surveillance, detection, and prevention, and developed many coronavirus detection systems. (2) Detected herd immune markers and initiated a free coronavirus antibody assay. 3 Set up medical centers: (1) Designated 32 military hospitals to receive COVID-19 patients, established 7 temporary mobile hospitals and allocated and renovated beds in local hospitals and clinics. (2) Allocated 8.8 billion rubles for constructing 16 multi-purpose infectious disease medical centers; Expanding and renovating the hospital ship “Irtysh” to treat other patients in the Far East. 4 Community epidemic prevention measures: (1) Paid vacation was extended from Mar 28 to May 11, and states of emergency were authorized. (2) Quasi-lockdown city: People were allowed to leave homes on limited occasions; Recreational places remained closed; Supermarkets, pharmacies and other places posted signs that queue 1.5 meters apart. (3) Banned on gatherings of more than 5,000 people; Introduced masks wearing and a passport system.
Stage 3: Restarting economic and social activities	1 Relaxed Quarantine measures and resumed economic activities: Since Jun, several states have begun to relax quarantine measures and gradually resumed commercial activities. Sao Paulo state gradually opened shopping malls, restaurants, bars, and other public places in 5 stages. 2 Medical measures: (1) Zero tariff on 34 drugs used for COVID-19 treatment. (2) A nucleic acid testing without getting out of cars was offered 200 tests a day in a shopping mall parking lot in Sao Paulo. (3) Closed the Anhembi square cabin hospital. (4) Conducted clinical trials of 9 coronavirus vaccines, developed in China, Russia, and other countries. 3 Brazil's Ministry of Health suspended the release of key data on the COVID-19 outbreak.	1 Removed of restrictive measures by grading and stages: (1) On June 12, Russian nationwide holiday covering all sectors ended, and gradually lifted restrictions. In stage 1, lifted restrictions on sports, small-scale trade and service entities while maintaining social distance; In stage 2, people were allowed to walk outside, large businesses, physical service industries and educational institutions were allowed to limited open; In stage 3, opened public places at social distance, fully opened trade, physical services and all educational institutions and catering businesses. (2) According to epidemic prevention situations, each federal government managed by different levels. (3) On Jun 9, Moscow lifted policy of self-isolation, electronic pass system and walking requirement. (4) On Sep 1, opened universities and schools, and organized the opening examination. 2 Implemented a national economic recovery plan which forces on achieving economic growth and reducing unemployment. 3 Relaxation of entry and exit prevention and control: Resumed outboard flights; Allowed Russian to go abroad for medical treatment, take care, work, and study, and allowed foreign citizens inboard.
Stage 4: COVID-19 second outbreak	1 Some states upgraded prevention and control measures: The Rio Grande do Sul state government banned beach stays, suspended celebrations and restricted on catering hours; A curfew was imposed across Parana state; The state of Santa Catarina reopened empty intensive-care beds, tightened monitoring of masks and banned crowds; Sao Paulo state entered the most stringent epidemic prevention red phase, restricted time to open supermarkets, pharmacies, gas stations and other essential places; Para state canceled Carnival celebrations, banned public gatherings of more than 10 people, closed bars; The Federal District of Brasilia imposed restrictive measures on non-essential business. 2 Vaccination measures: (1) A four-stage priority vaccination strategy. The first is health workers, older people aged over 75 or over 60 and living in nursing homes, and indigenous people; then, people aged 60 to 74; the third Stage three is people with comorbidities; The fourth stage is for teachers, soldiers, police, rescue workers, prison officials and prisoners. (2) On Jan 18, 2021, all of Brazil's 26 states and the Federal District began vaccination.	1 Strict prevention and control measures: (1) Since Oct 28, Russia from 23 to 6 to ban recreational activities and provide public catering services; Strengthened disinfection measures; Required masks in public places; Enhanced detection of coronavirus. (2) Tightened immigration rules: illegal immigrants had to leave Russia on their own or risk deportation. 2 Restarted medical centers: (1) Resumed the temporary mobile hospitals in Patriot Park and Leningrad Exhibition Hall operation. (2) Two new multi-purpose medical centers were built in Astrakhan Prefecture and Narimanov. 3 Vaccination measures: (1) Registered the world's first COVID-19 vaccine (Sputnik-V). (2) Since Oct 28, Russia from 23 to 6 to ban recreational activities and provide public catering services, and the formulation of the "Vaccine Passport" and international certification were promoted.

#### Epidemic cooperation in BRICS

BRICS countries have actively engaged in COVID-19 cooperation and reached consensus through bilateral and BRICS Summit. Firstly, health cooperation: sent medical experts, provided medical supplies, established an early warning mechanism for infectious diseases, and sharing experience in fighting COVID-19; Secondly, vaccine cooperation: carried out vaccine clinical testing, production, and supply cooperation, established vaccine research and development center; Thirdly, economic cooperation: The New Development Bank granted $1 billion in emergency loans to each BRICS member countries for purchasing of personal protective equipment and easing the economic recession. Table 4 summarizes cooperation measures among BRICS countries.

**Table 4:** Cooperation measures taken for COVID-19 among BRICS countries

Measures	Key elements
Health cooperation	1 Sent medical experts: (1) On Feb 5, 2020, seven Russian epidemiologists arrived in Beijing to help China contain the spread of the virus and develop a vaccine. (2) On April 11, China sent a 10-member expert team to Russia to exchange experience and provide guidance and training on prevention and control, diagnosis, and treatment. 2 Provided medical supplies: (1) In Feb 2020, Russia, India and South Africa donated masks and other medical supplies to China. The Brazil-China Association for the Promotion of Peaceful Reunification donated to China. (2) Since March, China has donated personal protective equipment, nucleic acid extraction reagents and other materials in batches to Russia, India, Brazil and South Africa. (3) On April 17, India committed to delivering nearly 100 million Hydroxychloroquine tablets to Russia for the treatment of COVID-19. (4) On May 23, Russia delivered the Coronavirus diagnostic test system to South Africa. 3 Established an early warning mechanism for infectious diseases: (1) On April 28, 2020, heads of BRICS Foreign Ministries held a special meeting on COVID-19 to support the joint establishment of an early warning mechanism for infectious diseases. (2) On Sep 9, 2021, the New Delhi Declaration of the 13th BRICS Summit endorsed the progress made in establishing an early warning system for the prevention of large-scale infectious diseases in BRICS countries to identify and predict future pandemics through institutional cooperation. 4 Shared experiences in fighting COVID-19: (1) China, South Africa, Russia, and Brazil held video conferences for COVID-19 prevention and control. (2) Experts in the design of Leishenshan Hospital in China, shared construction experience and helped South Africa improve the construction plan of anti-epidemic hospitals. (3) From March to April 2021, the National School of Governance of China offered courses on national governance and emergency management for middle and senior leaders of South African public organizations.
Vaccine cooperation	(1) China and Russia are working together to develop and produce COVID-19 vaccines; Russia and Brazil carry vaccine cooperation in three aspects: clinical testing, vaccine production, supply to Brazil and the whole Latin American market; The Russian vaccine “Sputnik V” manufactured in India, Brazil, and China. (2) On July 24, 2020, Brazil launched a clinical trial of a coronavirus vaccine from China, which vaccinated 9,000 volunteers within 90 days. (3) On Nov 17, 2020, according to the Moscow Declaration of the 12th BRICS Summit, we will work to ensure that vaccines, once available, are distributed in a fair, equitable, and affordable manner; We also encourage the BRICS Vaccine Research and Development Center to become operational as soon as possible. (4) The contribution made by BRICS countries in providing over a billion COVID-19 vaccine doses, including grants and donations, bilaterally, to international organizations and the COVID-19 Vaccines Global Access (COVAX) facility.
Economic cooperation	(1) In April 2020, New Development Bank established an emergency assistance facility, including US $5 billion for health and social security expenditures and US $5 billion to support economic recovery of member countries. (2) New Development Bank has issued three COVID-19 preparedness bonds and $1 billion in emergency loans to five member countries to purchase hospital beds, ventilators, and protective equipment for frontline medical workers, as well as to mitigate the economic downturn caused by the pandemic and lockdown.

### Results of the prevention and control measures of COVID-19 in BRICS countries

#### Results of the prevention and control measures in China

**Fig. 1:**
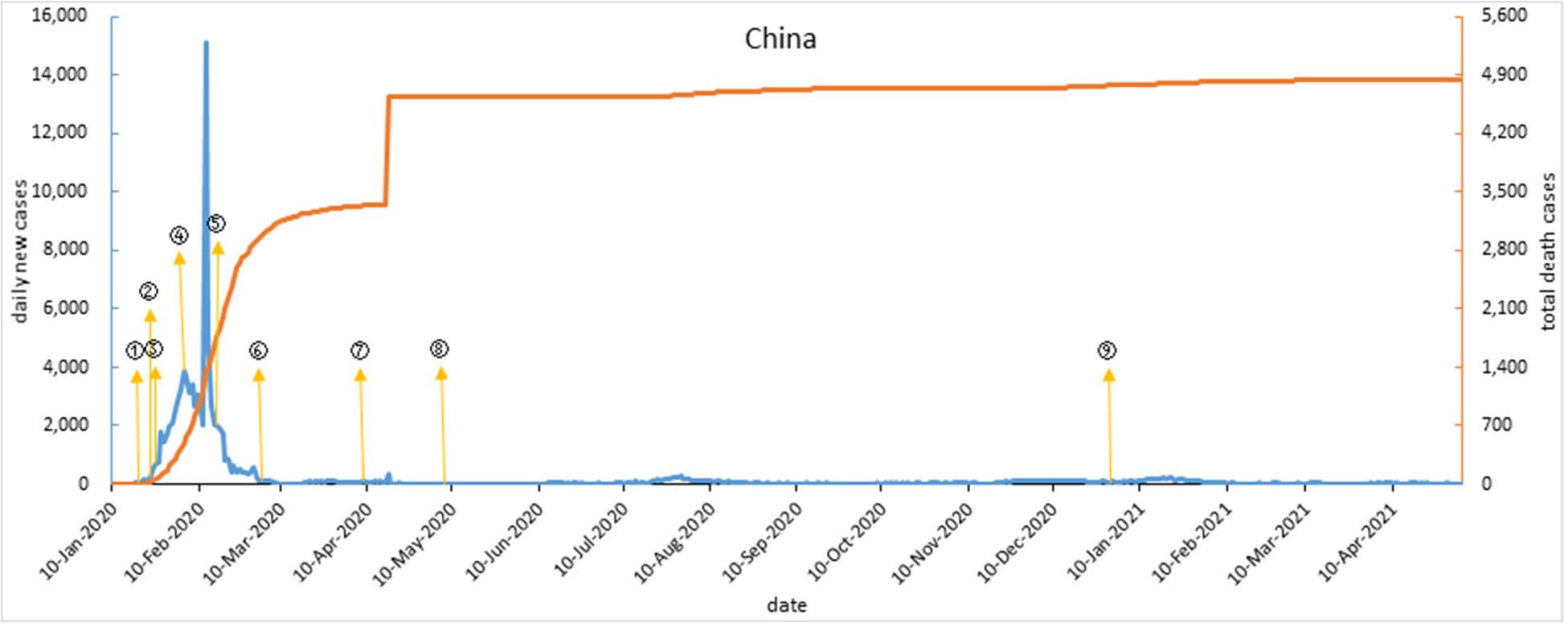
COVID-19 outbreak curve and timeline of implementation of major interventions in China. Note: ① On Jan 20, 2020, COVID-19 was included as a national class B infectious disease. ② At 10 o 'clock on Jan 23, lockdown Wuhan city and required all citizens to wear masks. ③ On Jan 25, all provinces with confirmed cases of COVID-19 launched a public health emergency response of level I. ④ On Feb 4, the Huoshen Mountain cabin hospital began to receive patients, organized pairing assistance from other provinces to cities in Hubei for treatment. ⑤ On Feb 16, all communities in Hubei Province implemented strict blockade management for 24 hours and launched a large-scale community screening. ⑥ On March 3, the COVID-19 diagnosis and treatment plan (trial seventh edition) was issued. ⑦ On April 8, Wuhan was unsealed. ⑧ On May 8, China's epidemic work moved from emergency status to normalization. ⑨ On Dec 31, COVID-19 vaccine was launched in China

Since the end of Jan 2020, China’s daily new cases have sharply increased and government launched a series of containment measures. On Feb 12, 2020, to revise diagnosis results, Hubei Province investigated previously suspected cases adding clinical diagnosis as the basis. After that, daily new cases reached a peak and then declined. On Mar 19, 2020, it was the first time reported no local cases. On April 8, 2020, Wuhan lockdown lifted, and epidemic improved. On May 8, 2020, local cases were stable, and China’s epidemic work shifted to preventing inbound cases and domestic resurgence [26]. During the normalization stage, there have been several sporadic domestic cases and local outbreaks, without a second epidemic wave. As shown in Fig. 1.

#### Results of the prevention and control measures in India and South Africa

**Fig. 2:**
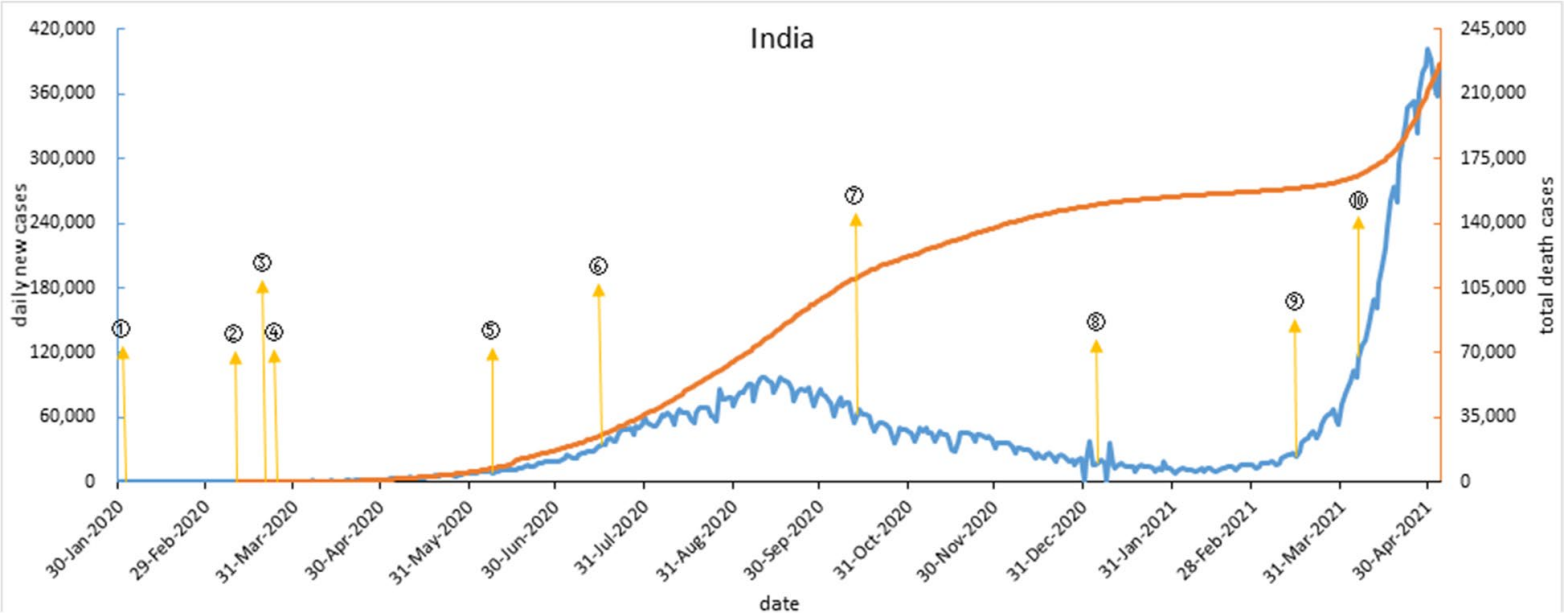
COVID-19 outbreak curve and timeline of implementation of major interventions in India. Note: ① On Feb 2, 2020, stopped the electronic visa service for Chinese and foreigners lived in China. ② On March 11, closed the border, passengers from severely affected countries would be under quarantine for 14 days. ③ On March 22, imposed a curfew calling on all Indians to stay indoors between 7 and 21. ④ Since March 25, a strict lockdown and quarantine was imposed across the country. ⑤ On June 8, opened places of worship, hotels, restaurants, shopping centers all across India and changed curfew time. ⑥ On July 17, resumed some international air travel. ⑦ On Oct 13, announced a new economic stimulus package about 730 billion rupees. ⑧ On Jan 6, 2021, launched a nationwide vaccination campaign. ⑨ On March 25, mass celebrations were banned during the traditional holiday. ⑩ Since April 6, New Delhi imposed a daily curfew, during curfew, only allowed shops providing essential services and vehicles used for emergency matters

India and South Africa have a very similar epidemic curve with a trough between two distinct peaks. On Jan 25, 2020, India implemented immigration control measures, kept daily new cases zero in the early period. By Mar 4, 2020, India’s confirmed cases had sharply risen after 15 Italian tourists were diagnosed. On Mar 25, 2020, India entered a five-stage blockade, with a slowly daily new cases increase and no spike. With “unsealed” policy began in June 2020, and enterprises gradually resumed work, the first wave began to climb, and peaked in Sep 2020. Then, infections declined slowly. By Feb 2021, daily new cases dropped below 20,000. But in early Mar 2021, the outbreak worsened with a slow confirmed case increase. Then COVID-19 explosion with economic activities restarted and population management relaxed. On April 11 2021, India became the world’s second-highest number of COVID-19 cases. As shown in Fig. 2.

**Fig. 3:**
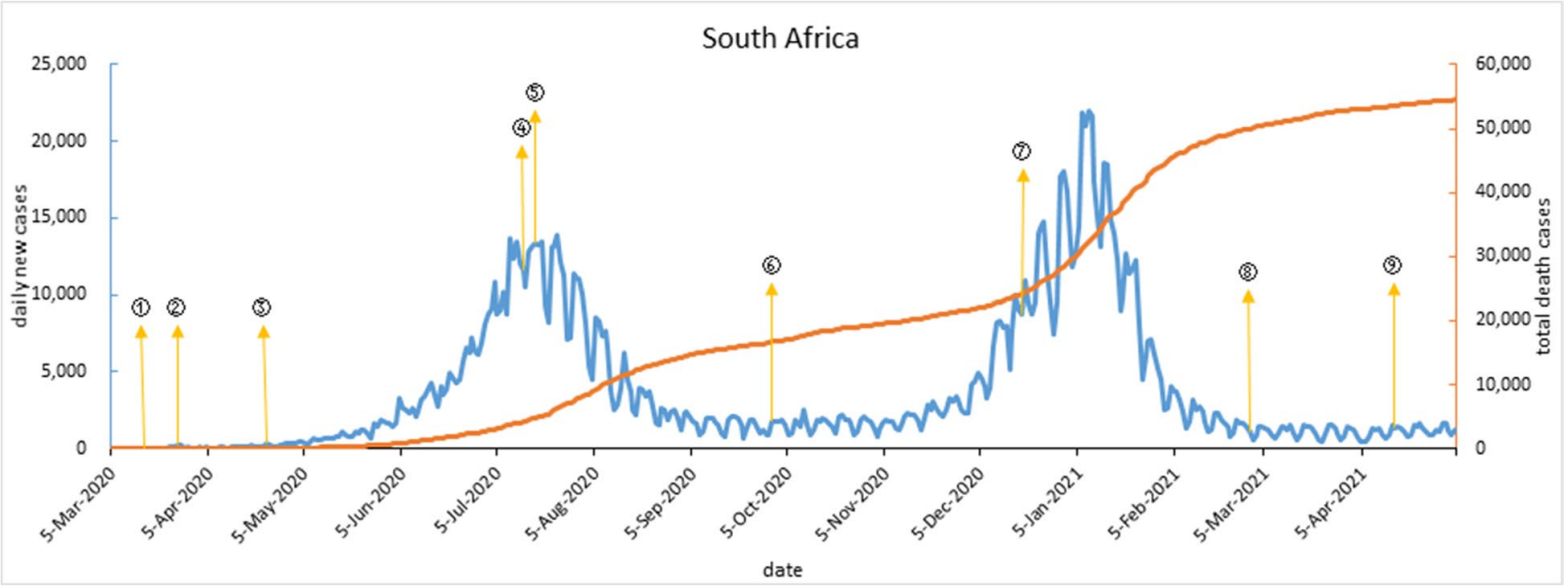
COVID-19 outbreak curve and timeline of implementation of major interventions in South Africa. Note: ① On March 15, 2020, suspended visa issuance to visitors from high-risk countries; Closed 35 land ports and 2 waterways; Strengthened airport monitoring measures. ② At midnight on March 26, South Africa began a 21-day "lockdown", and the lockdown level was set as Level 5, the strictest. ③ From April 23, gradually relaxed the level of lockdown order and allowed enterprises to resume work. ④ On July 13, a curfew was imposed from 14 to 4, alcohol bans were reintroduced and masks were required. ⑤ On July 17, 5,571 isolation sites were set up across the country. ⑥ On Oct 1, all bans on COVID-19 were fully lifted, borders were reopened and international travel resumed. ⑦ On Dec 18, the “lockdown” was upgraded from Level 1 to Level 3, and curfews, masks and alcohol bans were reinstated. ⑧ On Feb 28, 2021, upgraded the lockdown to Level 1, shortened curfews, permitted the sale of alcohol, and certain gatherings were allowed with conditions. ⑨ On April 16, electronic registration for the COVID-19 vaccine was opened to all

Since the first imported case from Italy was reported on Mar 5, 2020, daily new cases in South Africa have been slowly increasing. During a 21-day lockdown, daily new cases slowed down to within 100 cases. Since April 23, 2020, with the gradual easing of lockdown, daily new cases have re-increased and reached the peak of the first wave. The government restarted public health and social control measures, then the first wave gradually fell back. Affected by the mutant coronavirus 501Y.v2 [27], daily new cases re-increased in Nov 2020, reached the peak of the second wave in Jan 2021, then fell back rapidly. After the second wave outbreak, daily new cases had stabilized about 1,000 in May 2021. As shown in Fig. 3.

#### Results of the prevention and control measures in Brazil and Russia

**Fig. 4:**
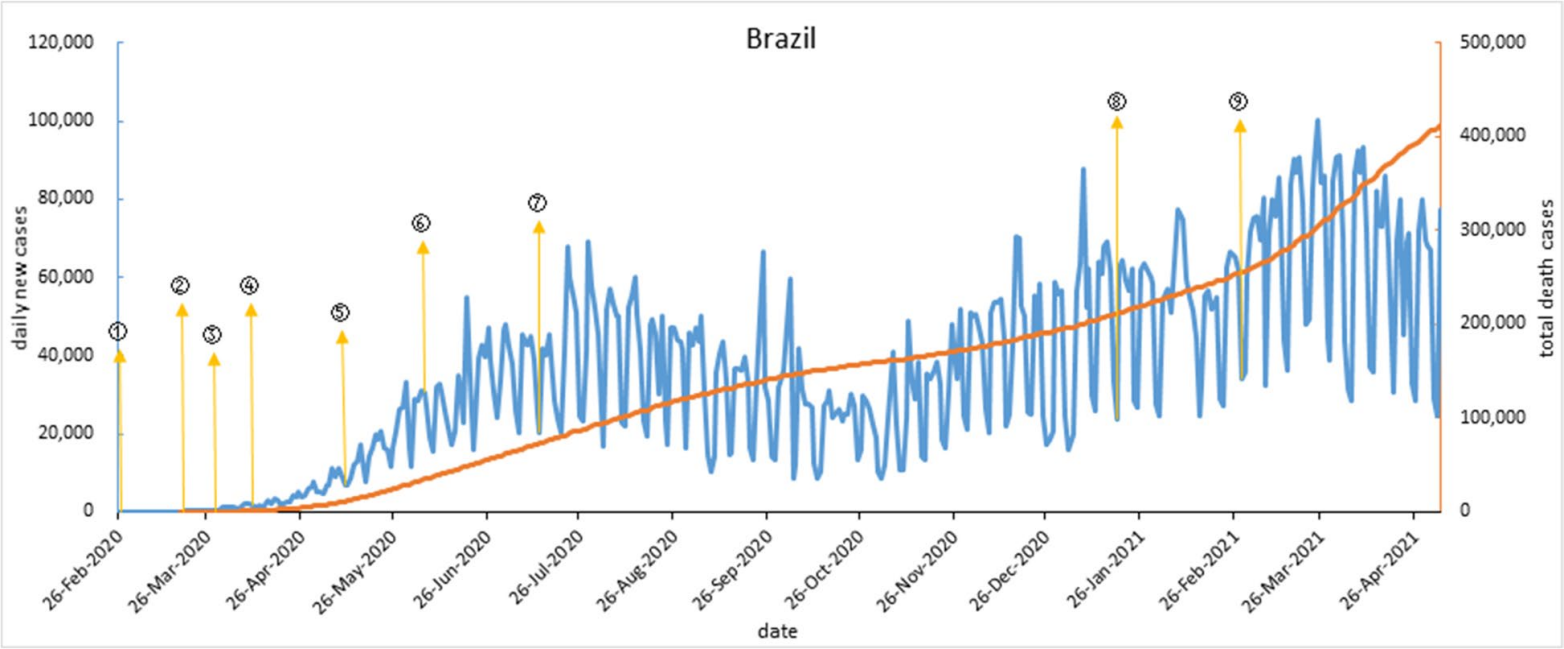
COVID-19 outbreak curve and timeline of implementation of major interventions in Brazil. Note: ① On Feb 27, 2020, closed elementary and middle schools. ② On March 20, Brazil entered into a state of public disaster. ③ On March 30, Brazil completely closed its borders; Encouraged people to wear masks. ④ On April 11, the Anhembi square cabin hospital in Sao Paulo began receiving patients. ⑤ On May 11, the government issued a decree stipulating 57 essential activities and services, including construction work and industrial production, to urge businesses to reopen. ⑥ On June 5, Brazil's Ministry of Health suspended the release of key data on the COVID-19 outbreak. ⑦ On July 13, zero tariffs on 34 drugs used for COVID-19 treatment. ⑧ On Jan 18, 2021, all of Brazil's 26 states and the Federal District began vaccination. ⑨ On Feb 28, 12 states and the Federal District of Brasilia upgraded prevention and control measures, with curfews imposed in seven of them. (Note: Data vacancy exists on Sep 23, Oct 2, Oct 18, Nov 5, 2020 and Feb 6 and Feb 8, 2021)

The trend of the epidemic curve in Brazil and Russia were similar, with two obvious epidemic peaks. Unlike India and South Africa, there was no effective drop between the two epidemic peaks, that is, after an epidemic peak, daily new cases were still higher than the previous one. Brazil confirmed its first case on Feb 26, 2020. More than half a month, confirmed cases slowly increased. When the first death was reported on Mar 17, 2020, COVID-19 in Brazil had gradually spread, and by Mar 20, 2020, whole country had entered a state of public disaster. On April 5, 2020, total confirmed cases in Brazil exceeded 10,000, making it the worst-affected country in South America. From early Mar to May 2020, when Brazil’s federal and local governments responded to COVID-19 with different strategies, infection was spreading. Since the beginning of June 2020, the epidemic has been gradually unsealed and economy has resumed, but the epidemic has worsened. And the second wave began in Nov 2020. In addition, daily new cases curve in Brazil shows a cyclical trend of five days increasing and two days decreasing, which coincides with Brazilian rest days. As shown in Fig. 4.

**Fig. 5:**
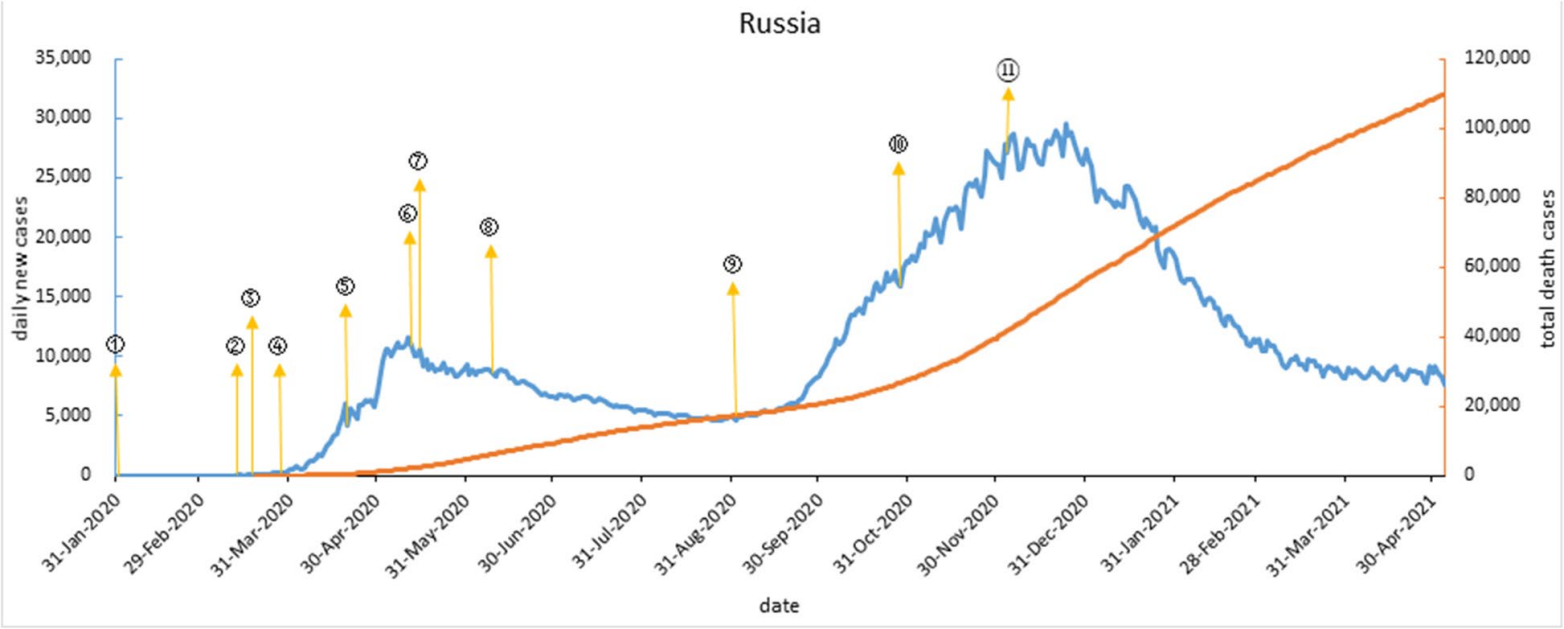
COVID-19 outbreak curve and timeline of implementation of major interventions in Russia. Note: ① On Jan 31, 2020, Russia closed its far eastern border with China, temporarily banned Chinese citizens from entering the country for private purposes. ② On March 13, suspended most scheduled flights to Italy, Germany, Spain and France and halted flights to and from China. ③ On March 18, banned foreign citizens entering Russia and required entry quarantine for 14 days. ④ Paid vacation extended from March 28 to May 11. ⑤ On April 20, a flurry of medical centers began to treat COVID-19 patients. ⑥ On May 12, Removed restrictive measures by grading and stages. ⑦ On May 15, the capital Moscow took the lead in launching free antibody testing voluntarily. ⑧ On June 9, Moscow lifted policy of self-isolation, electronic pass system and walking requirement. ⑨ On Sep 1, opened universities and schools and organized the opening examination. ⑩ Since Oct 28, Russia from 23 to 6 to ban recreational activities and provide public catering services. ⑪ On Dec 4, the vaccine was launched for all

On Jan 31, 2020, after two confirmed cases, Russia immediately closed border and successfully controlled import cases. In mid-Mar, confirmed cases began to rapidly increase. On May 3, 2020, daily new cases exceeded 10,000. On May 11, 2020, national paid vacation ended and restrictions were lifted in stages, with daily new cases a steady drop. On Sep 1, 2020, Russian schools opened, and daily new cases rebounded, with the second wave beginning. Restrictions were introduced in late Oct 2020 and a nationwide vaccination was launched on Dec 4, 2020. The second wave peaked in Dec 2020 and gradually subsided, with daily new cases stabilizing to around 8,000 by May 2021. As shown in Fig. 5.

#### The total confirmed cases, total deaths, and total deaths per million in BRICS countries

**Fig. 6:**
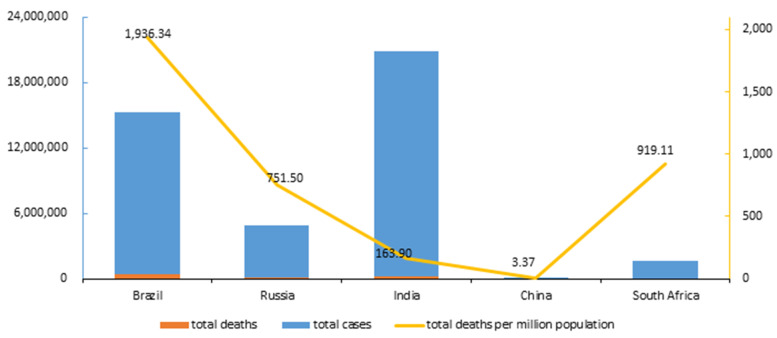
Total confirmed cases, total deaths, and total deaths per million in BRICS countries

China’s total confirmed cases have stabilized at 10,000, while India, Brazil, Russia, and South Africa have the highest confirmed cases in the world. Brazil’s total deaths were higher than India which had 5.8 million more confirmed cases. Total deaths per million China remained at 3.37 lower than India at 163.90, Russia at 751.50, South Africa at 919.11, and Brazil at the highest of 1,936.34. As shown in Fig. 6.

## Discussion

Based on the national health care system, population structure, and epidemic situations, BRICS countries took different interventions to combat COVID-19. China adhered to containment strategy, focusing on the whole transmission process; India and South Africa adopted intermediate strategies, taking different strategies at different epidemic stages; Brazil and Russia took mild mitigation strategies to ease pressure on health care systems.

Curitiba’s Declaration highlighted equity as a prerequisite for health and as an essential element of health promotion [28]. Many countries are facing insufficient health costs, vulnerable groups difficulty accessing health care, and inequity factors between countries. These health inequity factors accelerate disease development, especially in COVID-19. International solidarity and cooperation in fighting pandemics, conducting active international exchanges and cooperation, and building a Global Community of Health for All are effective to contain the spread of COVID-19 pandemic.

### Containment strategies

China’s prevention and control effect shows that containment strategies can have a positive impact on limiting the spread of COVID-19 [19]. Containment strategies focus on strict prevention and control of the Coronavirus transmission process, which requires extensive funding to support nucleic acid testing, medical treatment, and close contact screening. The Current health expenditure per capita in China in 2018 was only US$501.06 [29], less than half of the world average. However, China covered all costs of COVID-19 treatment, which was about US$3,542 for a confirmed patient [26]. In addition, all COVID-19 treating drugs were included in medical insurance, ensuring equitable access to medical treatment. China adheres to the principle of all those in need are treated, which reflects adherence to the supremacy of the people and life.

At the early stage, China carried out several epidemiological investigations and lockdown Wuhan city to limit the rapid spread of COVID-19 epidemic, and closely tracked and managed the “four categories of people”. Facing surge medical demand contradicted the lack of medical resources, China established cabin hospitals, organized pairing assistance from provinces to cities, and so on. Public ownership of hospitals and a philosophy of support from all sides when one side is in trouble ensured these measures’ implementation. For imported cases, the first point of entry and closed-loop management were implemented to reduce harm. Although in the normalized stage of epidemic prevention and control, there have been several clusters and sporadic cases, China has actively carried out epidemiological investigations and traced the source of confirmed cases, searched for close contacts, and delineated the risk level and control area scientifically and accurately, and all cluster cases have been effectively controlled.

Lockdown measures are a key part of containment strategies and are mostly implemented in early stages and large clusters. China, India, and South Africa all imposed lockdown measures, which effectively slowed virus spread. Early lockdown measures can slow down large-scale outbreaks, reduce cross-infection, and facilitate screening of patients. It could also reduce the burden on the health system.

### Intermediate strategies

The intermediate strategy is choosing containment strategy or mitigation strategy at different stages. And intermediate strategies are preferred in many less developed countries and countries with few medical resources. In general, Containment strategies were implemented in the early stages to prevent and control the epidemic, then implemented mitigation strategies were to safeguard the normal conduct of productive life. However, the timing of choosing to implement mitigation strategies needs to be scientifically determined based on the country’s situation.

India and South Africa both adopted containment strategies in the early stages of the domestic outbreak. Between Mar and June 2020, India implemented a five-stage blockade, with a ban on domestic and international travel, and daily new cases increased slowly. However, India’s poor account for 9.7%, they concentrate in slums, small living areas, densely populated populations [30], which can’t reach conditions for home isolation, coronavirus was rapidly spreading among the poor. In the first blockade, India implemented policies of closing factories and public transportation, which lacked attention to workers and led to many people returning home on foot, increasing movement and virus spread. Resumed work and production without the epidemic being controlled, the second wave has become more and more intense with the help of coronavirus variant. Affected by the mass gathering of religious activities and inadequate epidemic education, knowledge and implementation of COVID-19 prevention and control measures are not in place. In addition, India’s Current health expenditure per capita in 2018 was only US$72.83 [29], with inadequate primary health care and public health system [31, 32], inability to guarantee basic health equity. As for the low total deaths per million in India, the government recognized the lack of health resources, in the spreading stage implemented a five-stage blockade and handwashing with soap [31], effectively containing the outbreak. In addition, India is the 2nd largest populous country and the population structure is young with fewer basic diseases, which is strongly immune to the virus [33].

Thanks to the 21-day nationwide lockdown and preceded border closures, the curve of daily new cases in South Africa was demonstrably flattening [34], with the infection rate dropping to 4 percent. These measures delayed the epidemic peak and won time to prepare medical supplies and organize staff. Infectious disease is a major problem impacting the health of African people [35]. In COVID-19 close contact tracing and management, South Africa organized 10,000 members of Tuberculosis control teams to undertake the work. Unlike in containment strategy that implements nonessential not go out, South Africa lockdown measure still allowed social gatherings below 100 people which led to several funeral-gathering infections. Even so, unemployment still hit 30.1%, making the stop-production measures unsustainable. South Africa prematurely lifted the blockade and resumed production, which led to a surge of infected people and a second epidemic wave, which is consistent with the findings of American scholar Richard [36] that early withdrawal of interventions will lead to a rebound or resurgence of the epidemic. Refugees, international asylum-seekers, and undocumented migrants were excluded from policies and COVID-19 brought a disproportionate impact on them [37]. South Africa’s Current health expenditure per capita in 2018 was US$525.96 at a low level [29], which is another reason for the high death rate.

Resuming work is an urgent need, but a key reason for many countries’ COVID-19 rebound. It is also a turning point of intermediate strategy. When COVID-19 was not stabilized, both India and South Africa resumed work, and relaxed social restrictions, leading to the second wave. Therefore, it is important to choose the time to implement resumption of work policy according to the realities in their countries, as well as prevention and control policies such as social distancing, wearing masks, and personnel management.

### Mitigation strategies

Mitigation strategies focus on close contact tracing and critically patient treatment, supplemented by slight social restrictions and blockade. The goal is to keep COVID-19 slow spread and smooth epidemic peaks under maintaining a normal life.

Brazil’s federal and local governments were divided in responding to COVID-19. The federal government kept passive response and the leader believed that prevention measures must be carried out under normal production activities [38]. They only advocated wearing masks and closing schools. Rapid data sharing is the basis for control measures’ development and implementation during epidemic [39], but Brazil’s key epidemic data was suspended several times. Brazilian President Bolsonaro’s “passive response” was considered the biggest threat to COVID-19 [40]. Local governments made the main community-based measures, which caused a wide variation in measures across the country. Brazil’s medical system is based on universal free medical care, but faces poor infrastructure, a large gap of wealth, and a shortage of prevention materials. Like India, Brazil has many slums where the poor live on transient incomes, making it difficult to maintain long-term home isolation. Furthermore, due to mutated viruses, Brazil’s both Current health expenditure per capita and rate of deaths per million people were the highest among BRICS countries. Especially among the poor, more than half of deaths occurred in the intensive care units of public hospitals.

Russia advocated “mild epidemic prevention”, which was seeking a middle way between lockdown city and complete lifting of restrictions. In fact, Russia adopted typical mitigation strategies. Even during “quasi-lockdown city” with national paid vacation, gatherings to 5,000 people were restricted, and wearing masks mandatory began in May 2020. A Russian study concluded that a comprehensive blockade of marginal effect would have less than restrictions on food, drink, and public gatherings [41]. Until the second wave, the medical community and government agreed that strict restrictive measures were unnecessary, and COVID-19 in Russia was controllable. However, the epidemic grew to a high level. Russia is located on the Eurasian continent and connected to the Worst-hit areas at the early stage of COVID-19. Russia is a vast territory with a sparse population, and its population and economy are concentrated in the European part. COVID-19 also showed huge spatial disparities, with similar regional distributions of infections and GDP per capita. Russia followed universal health care in Soviet with a capable health system capacity, learned about Chinese and Italian experiences, established multiple application mobile cabin hospitals, and organized mass nucleic acid testing. These measures detected early cases, timely carried out medical treatments and kept a relatively low death rate.

Carrying out vaccination is important for both containment and mitigation strategies to establish an immune barrier. Vaccines are an important tool to help protect people against viruses and reduce disease severity. In mitigation strategies, it and population-controlled infection work together to establish an immune barrier. Compared with other countries’ efforts to develop, produce and acquire COVID-19 vaccines, Brazil did not actively carry out vaccine research and refused to accept COVID-19 vaccine assistance from Russia and China in the early stage.

### Health equity in BRICS

Health inequity accelerates COVID-19 spread. Meanwhile, COVID-19 got our attention on health equity, and we face an extraordinary opportunity to advance health equity. As “Buckets Effect” shows: water produced in a bucket depends on the shortest plank. A country’s epidemic control effectiveness depends on the weakest prevention and control measure. The world pandemic control effectiveness depends on the worst-affected country. Health equity in BRICS countries includes two aspects: health equity within countries, and health equity among BRICS countries.

According to the World Bank, Current health expenditure per capita in BRICS countries was below the world average of US$1,111.08 [29], and a few rich people used most medical services. It is urgent to improve the primary health care system and increase health care equity. Coronavirus is mainly spread by droplet transmission [42], which is accelerated in congregating populations and living conditions with unsafe social distances such as slums and prisons. With a high proportion of poor people in BRICS countries, the ability to guarantee health equity for medically vulnerable populations affects COVID-19 control [43].

Making intervention policies more inclusive and incorporating health equity practices into all intervention measures can improve health equity and fight the epidemic successfully. For example, South Africa’s single-room policy provided temporary shelters for homeless people and self-isolation places with unused houses and low investment, and China used government finance to cover all costs of COVID-19 treatment when the current health expenditure per capita was at a low level. Two examples use policy and government finance to fill shortcomings of epidemic prevention and control.

It is significant to keep health equity between countries in a worldwide pandemic that would damage health, economic and social development. Health inequity among BRICS countries is impacted by “vaccine nationalism” that banning exports of vaccines and key materials, artificial creating “immunization gap”, politicizing the pandemic that blaming other countries for COVID-19 [8], restricting the export of medical supplies, and so on. These policies negatively affected health equity, cooperation, and mutual trust among BRICS countries in fighting COVID-19 which are not conducive to stem the worldwide pandemic.

### Epidemic cooperation in BRICS

The COVID-19 prevention and control of the pandemic is not a matter of a single country. Virus knows no border, humans are a community of destiny, countries in the world are closely linked and interconnected. Only when the global pandemic is effectively controlled can they protect themselves, which requires international epidemic cooperation to achieve effective COVID-19 control. Foreign policy offers a means to achieve health equity and better health [44].

COVID-19 outbreak intensified international health cooperation: send medical experts, provided medical supplies, shared experience in fighting COVID-19. Health cooperation provides material support for COVID-19 prevention and control. Personal protective equipment and medical equipment effectively reduced the infection rate of health workers and the death rate of COVID-19 patients. Vaccine cooperation led by countries that own vaccines, including vaccine research, development, production, distribution, and donation has contributed to promoting vaccine equity, reducing infection and morbidity rates, and realizing herd immunity at an early date.

However, there are still some problems with BRICS cooperation. First, BRICS is a partnership, which is not binding on the actual behavior of each country; secondly, COVID-19 has caused obstacles to political trust and economic exchanges; moreover, each country has different COVID-19 response strategies, needs of medical resources and international cooperation.

Cooperation is the right choice to promote health equity and cope COVID-19 outbreak. In cooperation, BRICS countries should discard prejudice and seek common ground while reserving differences. Furthermore, BRICS Strategic Partnership should not only stay in exchanges and consensus. It should take concrete actions and produce concrete results to work together against the COVID-19 pandemic. BRICS countries continue to deepen epidemic cooperation under mutual trust and public health challenges, abandon the “nationalism” vaccine, promote to build the BRICS health community and a Global Community of Health for All [45].

## Conclusion

BRICS countries have implemented different interventions to limit the spread of coronavirus. China adopted containment strategy, South Africa and India adopted intermediate strategy combining containment strategy with mitigation strategy, and Brazil and Russia adopted mitigation strategy. The number of daily new cases in China is lower than in the other four countries. These results suggest that containment strategies are more effective than intermediate and mitigation strategies in limiting the spread of COVID-19. Especially when strict containment strategies are implemented early in the outbreak, but premature relaxation of restrictions may lead to a rebound and accelerated the spread of COVID-19. It is a good choice to combat COVID-19 by improving the inclusiveness of intervention policies, deepening BRICS epidemic cooperation, and increasing health equities.

## Data Availability

The datasets analyzed during the current study are available in the Johns Hopkins Coronavirus Resource Center repository, [https://coronavirus.jhu.edu/]; World Health Organization, [https://www.who.int/data#dashboards]; and National Health Commission, PRC [http://www.nhc.gov.cn/xcs/yqtb/list_gzbd.shtml (Webpage in Chinese)].
